# Prolonged Hypocalcemic Effect by Pulmonary Delivery of Calcitonin Loaded Poly(Methyl Vinyl Ether Maleic Acid) Bioadhesive Nanoparticles

**DOI:** 10.1155/2014/932615

**Published:** 2014-02-20

**Authors:** J. Varshosaz, M. Minaiyan, M. Forghanian

**Affiliations:** ^1^Department of Pharmaceutics, School of Pharmacy and Novel Drug Delivery Systems Research Centre, Isfahan University of Medical Sciences, P.O. Box 81745-359, Isfahan 81746-73461, Iran; ^2^Department of Pharmacology, School of Pharmacy, Isfahan University of Medical Sciences, Isfahan 81746-73461, Iran

## Abstract

The purpose of the present study was to design a pulmonary controlled release system of salmon calcitonin (sCT). Therefore, poly(methyl vinyl ether maleic acid) [P(MVEMA)] nanoparticles were prepared by ionic cross-linking method using Fe^2+^ and Zn^2+^ ions. Physicochemical properties of nanoparticles were studied *in vitro*. The stability of sCT in the optimized nanoparticles was studied by electrophoretic gel method. Plasma calcium levels until 48 h were determined in rats as pulmonary-free sCT solution or nanoparticles (25 *μ*g**·**kg^−1^), *iv* solution of sCT (5 *μ*g**·**kg^−1^), and pulmonary blank nanoparticles. The drug remained stable during fabrication and tests on nanoparticles. The optimized nanoparticles showed proper physicochemical properties. Normalized reduction of plasma calcium levels was at least 2.76 times higher in pulmonary sCT nanoparticles compared to free solution. The duration of hypocalcemic effect of pulmonary sCT nanoparticles was 24 h, while it was just 1 h for the *iv* solution. There was not any significant difference between normalized blood calcium levels reduction in pulmonary drug solution and *iv* injection. Pharmacological activity of nanoparticles after pulmonary delivery was 65% of the *iv* route. Pulmonary delivery of P(MVEMA) nanoparticles of sCT enhanced and prolonged the hypocalcemic effect of the drug significantly.

## 1. Introduction

Calcitonin (also known as thyrocalcitonin) is a polypeptide hormone of 32 amino acids, with a molecular weight of 3454.93 Daltons (approximately 3.5 kDa). Its structure is comprised of a single alpha helix. CT hormone is produced in humans primarily by the parafollicular cells of the thyroid, and in many other animals in the ultimobranchial body. It acts to reduce blood calcium (Ca^2+^), through opposing the effects of parathyroid hormone (PTH) [1–3]. Calcium plays an important role in most of cell activities. It contributes in muscles contraction and materials metabolism intracellular, and in bones, blood coagulation, and the transition of nervous signals extracellular [4, 5]. CT is found widely throughout different species such as fish (salmon), reptiles, birds, and mammals, while there are several differences in amino acid composition of CT from different sources which is associated with different potencies. Salmon calcitonin (sCT) is widely used because it is the most potent CT available with good tolerability. Currently, it is produced either by recombinant DNA technology or by chemical peptide synthesis [6–8]. This hormone acts both directly on osteoclasts, resulting in inhibition of bone resorption, and directly on chondrocytes, attenuating cartilage degradation and stimulating cartilage formation [9]. CT lowers blood Ca^2+^ levels in three ways: (1) inhibiting Ca^2+^ absorption by the intestines, (2) inhibiting osteoclasts activity and activating osteoblasts in bones, (3) inhibiting renal tubular cell reabsorption of Ca^2+^ allowing it to be secreted in the urine. However, the effect of CT that mirrors those of PTH is inhibition of phosphate reabsorption by the kidney tubules [10–14]. It seems that CT hormone has important effects on bone amendment, especially spinal column, then sCT is used for the treatment of postmenopausal osteoporosis, hypercalcaemia, Paget's disease, bone metastases, and phantom limb pain [15].

Bioavailability of sCT following subcutaneous and intramuscular injection in humans is high and similar for the two routes of administration (71% and 66%, resp.). Plasma protein binding is 30% to 40%, following parenteral administration of 100 IU sCT and its peak plasma concentration lies between about 200 and 400 pg mL^−1^ [16].

Currently, sCT from salmon species is commercially available in the form of injection and intranasal spray (Miacalcin). Data on the bioavailability of Miacalcin that is rapidly absorbed from nasal mucosa shows great variability with an average bioavailability of 3% compared to injection form [17, 18]. The maximal sCT plasma concentration is reached within 31–39 min following nasal administration of sCT solution. Moreover, sCT elimination half-lives of this way are 43 minutes [19].

Due to its short half-life (approximately 15–20 min) after parenteral administration, frequent administrations are required to maintain its pharmacological effects [19]. Moreover, in effective inhibition of the clinical symptoms of metabolic bone disorders such as osteoporosis or Paget's disease, a relatively high dosage of sCT is administered. Calcitonin may also be as an alternative for patients who cannot take the bisphosphonate drugs. However, the daily injection of sCT has poor patient compliance for long-term therapy. Therefore, controlled release delivery systems of this drug have attracted the interest of pharmaceutical researchers. Currently, the most common delivery route to administer sCT is through intramuscular or intravenous injections. Various researchers have studied different routes to deliver sCT more effectively and to decrease frequent administrations, including oral [20, 21], nasal [22, 23], vaginal [24], and rectal [25, 26] delivery. These systems are designed to protect the polypeptide against the biological barriers.

Petersen et al. [27] attempted to enable intestinal sCT absorption in rats using the mild surfactant, tetradecyl maltoside (TDM) as an intestinal permeation enhancer. TDM caused an increased absolute bioavailability of sCT in rat colon from 1.0% to 4.6%, whereas no enhancement increase was observed in ileal and jejunal instillations.

In another study, sCT-containing microspheres were designed for colonic delivery by applying a pH-sensitive polymer Eudragit P-4135F which showed a four-fold increase of the area above the curve of calcium blood levels compared to levels reached by sCT solution after 8–12 h [20].

Another polymer that was used in the production of sCT microcapsules was biodegradable poly(DL-lactic acid) [28]. Poly(lactide-co-glicolide) (PLGA) microspheres [29, 30], polyisobutylcyanoacrylate nanoparticles [31], and poly(ethylene glycol)-terephthalate (PEGT)/poly(butylene terephthalate) (PBT) [32] are also among the other reported biodegradable polymers used for controlled and sustained delivery of sCT. Absorption enhancers such as hydroxypropyl- and dimethyl-beta-cyclodextrins [33], the bile salts, chitosan, and loryl carnitin were evaluated for enhancing absorption of sCT from nasal route in rats [34].

Moreover, nanoparticles of glycol chitosan and its thiolated derivative resulted in a pronounced hypocalcemic effect for at least 12 and 24 h and a corresponding pharmacological availability of 27 and 40%, respectively, by improving the pulmonary mucoadhesive properties [35]. Relative lung bioavailability of these nanoparticles ranged from 11% to 18%. All formulations were prepared by spray drying powders containing sCT, human serum albumin, mannitol, and citric acid/sodium citrate [36].

Pulmonary administration of elcatonin loaded in chitosan-modified PLGA nanospheres resulted in more slowly elimination of drug from the lungs and reduced blood calcium levels to 80% with prolonged pharmacological action for 24 h which was significantly longer than that by chitosan-unmodified PLGA nanospheres. The absorption-enhancing effect may have been caused by opening the intercellular tight junctions [37].

The sCT powder suitable for inhalation was prepared using a spray-drying process which contained chitosan and mannitol as absorption enhancer and protecting agents.

Although some studies have been on the pulmonary delivery of sCT, there is not any pulmonary marketed dosage form of this drug yet. Consequently, further studies on more versatile drug delivery systems of this drug are necessary to increase its bioavailability. In the present study, bioadhesive nanoparticles of sCT were developed for pulmonary delivery using P(MVEMA) as a mucoadhesive polymer [39, 40] to increase the absorption of the drug in lungs mucosa.

Poly(methyl vinyl ether-alt-maleic acid) is a water-soluble poly anion having molecular masses ranging from 20 to 1980 kDa (average 522.45 kDa). The nanoparticles made from anhydride form of this copolymer have shown strong bioadhesive interactions with components of the gut mucosa [41–43].

At the moment there is not any pulmonary dosage form of sCT in the market and the present intranasal and intramuscular forms have only 3 and 5% bioavailability, respectively, with half-lives of less than 1 hour. The low bioavailability and short half-life of this drug have caused frequent need for drug administration which increases the cost of the therapy and reduces the patient compliance. For these reasons, the aim of the present study was to produce inhalable nanoparticles of sCT so as to increase the duration of hypocalcemic effect of this drug and enhance its bioavailability by its delivery through the pulmonary route of administration.

## 2. Materials and Methods

### 2.1. Chemicals and Reagents

sCT (Limhamn, Sweden) was used as the active ingredient. Poly(methyl vinyl ether-alt-maleic acid), mucin, and Coomassie Brilliant Blue G-250 dye were purchased from Sigma Chemical Company (USA). Zinc sulfate, iron chloride, ethanol, methanol, hydrochloric acid, and NaOH were purchased from Merck Chemical Company (Germany). Orthophosphoric acid 85% w/v was from Panreac (Spain).

### 2.2. Preparation of sCT Loaded P(MVEMA) Nanoparticles

A two-level factorial design was used for preparation of sCT loaded nanoparticles. Three different variables including stirrer rate (400 or 1000 rpm), cross-linking ion type (Fe^+2^ or Zn^+2^), and curing time (5 or 20 min) were studied and 8 different formulations (Table 1) were designed.

Nanoparticles were prepared by an ionic cross-linking method of P(MVEMA) [42] to obtain optimized nanoparticles with suitable particle size, zeta potential, drug loading efficiency, drug release efficiency, and mucoadhesive properties. In a typical procedure, 250 mg of P(MVEMA) was dissolved in 5 mL of deionized water at room temperature on a magnetic stirrer (IKA-WERKE, model RT 10 Power, Japan) with the speed of 450 rpm, and after 20 minutes when it dissolved completely, the pH of the solution was adjusted on 7.4 by adding NaOH. Afterwards, 2.5 mg of sCT was added to this solution while being stirred. In the next step, a 5% solution of FeCl_2_ or ZnSo_4_ (125 mg of the salt in 2.5 mL of deionized water) was prepared. The solution of the salts was added dropwise to the polymer/drug solution by an insulin syringe in various stirring rates (according to Table 1) while being stirred at different speeds. An overview of the investigated formulations is presented in Table 2.

A run involved the corresponding combination of levels to which the factors in the experiment were set. All experiments were carried out in triplicate. The effects of the studied variables on the responses were then analyzed by the Design Expert statistical software (version 7.0.0, Stat-Ease, Inc., Minneapolis, MN, USA) to obtain independently the main effects of these factors, followed by the analysis of variance (ANOVA) to determine which factors were statistically significant.

### 2.3. Preparation of Bradford Reagent

The Bradford protein assay is a colorimetric or spectroscopic analysis procedure for determining the concentration of protein in solution. It involves the binding of dye Coomassie Brilliant Blue G-250 dye to proteins in acidic solution. The maximum absorbance for this solution of Coomassie Brilliant Blue G-250 dye shifts from 465 nm (red or cationic unbound form) to 595 nm (blue or anionic bound form) when binding to protein occurs. For assay of protein concentration, maximum absorbance of sample solutions was measured in *λ*_max⁡_ = 595 nm and protein concentration of samples was determined by standard curve of special protein. The Bradford assay is linear over a short range, typically from 0 to 2000 *μ*g mL^−1^ [43, 44]. In the present paper on the preparation of Bradford solution (5x), briefly, 125 mg of dark purple powder of Coomassie Brilliant Blue G-250 was dissolved in 62.5 mL of 96% ethanol or methanol on stirrer, and 125 mL orthophosphoric acid 85% w/v was added to this solution. The resulting solution was diluted with water to a final volume of 250 mL while the pH was less than 4.

For measuring the protein concentration of the samples taken from the medium of release test and also in measuring drug loading efficiency in the nanoparticles, 200 *μ*L of Bradford reagent (5x) was vortexed with 800 *μ*L of each sample, after about 5 minutes and samples absorbance was measured spectrophotometrically (RF-5301 PC, Shimadzu, Kyoto, Japan) at room temperature in *λ*_max⁡_ = 595 nm [45]. Blank samples were prepared by adding 800 *μ*L of deionized water to 200 *μ*L of Bradford reagent.

### 2.4. Standard Curve of sCT

Solutions with different concentrations of 2.5, 7.5, 15, 22.5, 30, 45, 60, and 75 *μ*g mL^−1^ of sCT were prepared by dilution of a 100 *μ*g mL^−1^ stock solution of this drug in deionized water. Then, the absorbance of each solution was measured spectrophotometrically at *λ*_max⁡_ = 595 nm by Bradford method. This procedure was repeated three times for each concentration and in three consecutive days.

### 2.5. Particle Size and Zeta Potential Determination

The mean particle size (z-average) and zeta potential of sCT nanoparticles were determined by Zetasizer (Zetasizer 3600, Malvern Instrument Ltd., Worchestershire, UK) at 25°C. All particle size measurements were performed in deionized water using a He-Ne laser beam at 658 nm with a scattering angle of 130° without dilution.

### 2.6. Entrapment Efficiency Determination

The entrapment efficiency percent (EE%) was determined after centrifugation (Eppendorf AG 23331, model 5430, Hamburg, Germany) of nanoparticles dispersion using Eppendorf tubes (Amicon Ultra, Ireland, and cut-off 10000 Da) by measuring the concentration of free drug in aqueous medium. For this purpose, 1 mL of the drug loaded nanoparticles was centrifuged at 10000 rpm for 15 min; then, the concentration of free drug in the filtrate was measured after adding Bradford reagent at *λ*_max⁡_ = 595 nm. The concentration of loading drug was calculated indirectly by calculating the difference between the initial concentrations of the drug used (2.5 mg/7.5 mL) and the concentration of free drug in the aqueous medium by Bradford method. The EE% was calculated using

(1) Drug  loading  efficiency  or  EE%  =Drugtotal−DrugfiltrateDrugtotal×100.

To ensure that the drug has not been absorbed by the filter, a control solution of the drug with similar concentration of drug in the nanoparticles was prepared and was centrifuged in the same time and speed as mentioned earlier. Then the concentration of free drug was determined by the same method as nanoparticles. The recovery percent of the drug was 99.4% which showed that almost no important absorption of the drug has happened.

In the present study, the indirect method was selected to determine the amount of drug entrapped in nanoparticle suspension as the direct methods for determining nanoparticle EE are always complex and time-consuming. For this purpose after separating the free drug from the loaded nanoparticles by centrifugation, the nanoparticles were soaked in aqueous medium and the dispersion was stirred at 500 rpm for 24 hours. Then the amount of released drug was measured which was in good agreement with the indirect method. Therefore, for saving time in all other experiments the indirect method was used for measuring the drug loading efficiency.

### 2.7. *In Vitro* Release of sCT from Nanoparticles

Drug release profiles were monitored in phosphate buffer saline (PBS, 0.01 M, pH 7.0) at 37°C. 1.5 mL of nanoparticles dispersion was placed in dialysis membrane bags (Mw cutoff 12000 Da, Membra-Cel, Viskase, USA). Prior to the release experiment a dialyzing step was carried out by placing dialysis bag in aqueous medium for about 0.5 h to separate free drug. After that, the bags were suspended in a beaker containing 7.5 mL of PBS under stirring at a speed of 500 rpm. At appropriate time intervals (0.5, 1, 2, 5, 20, and 24 h) the concentration of sCT released in the medium was determined after adding Bradford reagent by UV spectrophotometry method at *λ*_max⁡_ = 595 nm. Blank samples were tested by the same procedure. Release efficiency within 24 h (RE_24_%) was used to compare the release profiles and was calculated using

(2) RE24%=∫0ty·dty100·t×100.

### 2.8. Mucoadhesion Measurement of Nanoparticles

Briefly, a stock solution (1 mg mL^−1^) of mucin was prepared with acetate buffer (pH 4.5). One mL of the nanoparticles dispersion (containing 50 mg mL^−1^) and 2.5 mL of mucin solution were mixed, vortexed, and shaken at 37°C for 3 h. The ratio of the nanoparticles to mucin was 20 to 1. Then 1 mL of this solution was centrifuged and the UV absorbance of the centrifuged solution was measured after adding Bradford reagent by UV spectrophotometry method at *λ*_max⁡_ = 595 nm. The concentration of mucin adsorbed to the nanoparticles was determined by the standard curve of mucin and the difference between the total amount of the mucin used and the free mucin after centrifuging was calculated.

To plot the standard curve of mucin, specific concentrations (100, 200, 300, 400, and 500 *μ*g mL^−1^) of mucin in acetate buffer (pH 4.5) were prepared by diluting the stock solution (1000 *μ*g mL^−1^) and their absorbance was measured at *λ*_max⁡_ = 595 nm by adding Bradford reagent. All steps were carried out using the same method as sCT standard curve.

### 2.9. Morphology Study

The image of the smallest and the largest sCL loaded nanoparticles was characterized by scanning electron microscope (SEM) (ZEISS Company, containing EBSD system, Germany). The nanoparticles were primarily coated with a thin layer of Au/Pd after they were mounted on aluminum stubs and then were examined using an SEM instrument.

### 2.10. SDS-PAGE Assay

For evaluation the stability of sCT in optimized formulation during production and release test after 24 h SDS-page or gel electrophoresis method using precast gradient gels (20% Tris-HCl/glycine, Bio-Rad, USA) was used which separates proteins based on their molecular weight range. Appropriate volume (10–20 *μ*L) of unstained molecular weight protein marker (SMO431, Thermo 26610, Fermenta, Germany) containing proteins with molecular weights such as 116.0, 66.2, 45.0, 35.0, 25.0, 18.4, and 14.4 kDa; a sample taken from the release dialysis solution of the optimized nanoparticles after 24 h; a blank sample taken from the 24 h release medium of the same nanoparticles formulation but without drug; and a standard sample of sCT solution were pipetted into the SDS-page gel. The mixture of 10 *μ*L of each sample and 10 *μ*L of Laemmli sample buffer (62.5 mM Tris-HCl, pH 6.8, 25% glycerol, 2% SDS, 0.01% Bromophenol blue) with 8% *β*-mercaptoethanol was used before running the gel and then it was floated 5 minutes in the boiling bath prior to loading. Afterward, the loaded gel was placed in electrophoretic tank (Bio Rad, USA) and electrophoresed for 2 h at 100 V and then stained with Coomassie blue to visualize the protein bands. After 2 h it was rinsed and placed in destaining solution for 4 h. This step was repeated several times until area of the gel was completely transparent, and the bands were clearly visible.

### 2.11. *In Vivo* Evaluation of sCT Loaded Nanoparticles

#### 2.11.1. Animals

Male Wistar rats, 9 weeks old (weighing 185–330 g) from the animal house of the School of Pharmacy and Pharmaceutical Sciences of Isfahan University of Medical Sciences, were used to study the pharmacological effects of pulmonary administered sCT nanoparticles. The animals were fasted for 12 h (overnight) but had free access to water immediately before the experiment. All animals were maintained under conventional housing conditions (temperature 22 ± 2°C, relative humidity 55 ± 5%, fresh food, and in a 12:12 h light/dark cycle). Before drug administration, rats were weighed accurately for calculation of test doses. The animal study was approved by the guideline of the ethical committee of Isfahan University of Medical Sciences.

#### 2.11.2. Pulmonary and Intravenous Administration

*In vivo* pharmacological studies were performed on 28 rats. The rats were randomly divided into 4 groups each containing 7 rats. Initially, they were anesthetized by inhalation of ether for a brief period of 10–20 s. The mean sCT dose for pulmonary administration was considered to be 25 *μ*g kg^−1^ [36] and for intravenous (iv) administration it was considered as much as 5 *μ*g kg^−1^ to compare the effectiveness of the prepared sCT nanoparticles with the free drug that was used as *iv* or pulmonary. The sCT solution for *iv* injection was prepared by dissolving 50 *μ*g mL^−1^ of sterile distilled water. Then, the sCT solution (group 1) was injected into the lateral tail vein located on either side of the tail with 5 *μ*g kg^−1^ sCT in a single dose as *iv* administration. Animals were placed on a rechargeable heat pack or circulating warm water pad to keep them warm during anesthesia. Under the same conditions, sCT solution was administered pulmonary in the dose of 25 *μ*g kg^−1^ (group 2). Rats of group 3 were administered blank nanoparticles dispersion as negative control group and the fourth group received the optimized sCT nanoparticles dispersion with the dose of 25 *μ*g kg^−1^ according to the loaded sCT. Pulmonary drug administration to the lungs of anesthetized rats was done directly in a single dose using a 1A_1B microsprayer (Penncentury, USA) device that generates an aerosol by an atomizer-attached syringe. This device avoids surgical manipulation of animals and allows for a much deep and uniform deposition of the spray droplets in the lung. Anesthetized animals were placed in supine position on a 45° slanted support. After the administration, the rats were maintained in a headup position at an angle of 90° to horizon for 30 s.

#### 2.11.3. Measurement of Plasma Calcium Levels

The hypocalcemic effect of the sCT on the treated groups of rats was studied. Blood samples of approximately 250 *μ*L were collected via the retro-orbital sinus vein after anesthetizing rats with ether, by heparinized capillary at different time points until 24 h after treatment. At time zero and then at 0.5, 1, 3, 12, and 24 h after drug administration, blood sampling was carried out to determine the calcium concentration. The plasma was separated by centrifugation at 3000–4000 rpm speed for 10 minutes and total plasma calcium concentrations were estimated using a commercial photometric available calcium kit (Pars Azmon calcium commercial kit for calcium liquid test (CPC), Iran). The assay was carried out with 2 *μ*L of plasma after adding 1000 *μ*L of the mixture of two reagents of the kit. The absorbance of the plasma samples was measured at *λ*_max⁡_ = 570 nm immediately by a UV-VIS spectrophotometer (UV mini-SHIMADZU, model 1240CE, Japan) and then changed to concentration using a standard curve. All assays were run as duplicates with a standard curve for each assay.

The 48 h area under the reduction percentage of blood calcium level-time (h) curve (AUC_0-48_) (3) was used for comparison of blood calcium lowering effect of sCT developed nanoparticles with *iv *route of administration and pulmonary delivery of sCT solution:

(3) AUC0−t=∫0ty dt

Pharmacological activity (PA) of sCT nanoparticlesadministered from the pulmonary route was calculated from (4):

(4) Pharmacological  activity   =dose  of  iv  solution  of  sCT×AUCof  pulmonary  sCT  nanoparticlesdose  of  pulmonary  sCT  nanoparticles×AUC  of  iv  solution  of  sCT  ×100.

All the *in vivo* data were analyzed using the one-way analysis of variance (ANOVA) followed by the LSD post hoc test (SPSS 11.5, USA). *P* values less than 0.05 were considered significant for all statistical tests.

## 3. Results and Discussion

### 3.1. Particle Size

The results of physicochemical properties of 8 different formulations of nanoparticles loaded with sCT are shown in Table 3.

Particle size of the nanoparticles changed between 166.5 and 553.8 nm (Table 3). The pulmonary delivery of nanoparticles may be challenged by a number of physicochemical and physiological parameters that influence the inhalation deposition in the lungs. Small particles in the size range of 0.1–1.0 *μ*m are inspired in the alveoli but also are exhaled without being deposited significantly [46]. Nebulization of nanoparticles suspension as aerosol droplets [47] or microencapsulation of them as dry powder inhalers [48] that are appropriate for pulmonary administration are reported to enhance the handling and delivery of nanocarriers.

**Figure 1:**
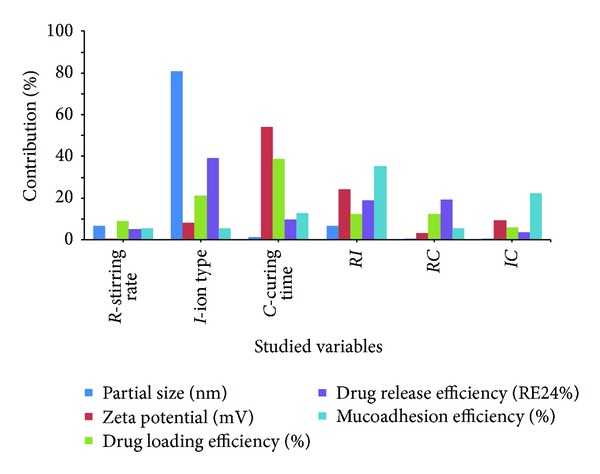
Contribution of different effective factors on the particle size, zeta potential, drug loading efficiency, drug release after 24 h, and mucoadhesion efficiency of sCT loaded P(MVEMA) nanoparticles.

Analysis of the results of Table 3 by Design Expert software using a two-level factorial design indicated that the most effective parameter on the particle size of the sCT nanoparticles was the ion type (Figure 1).

**Figure 2:**
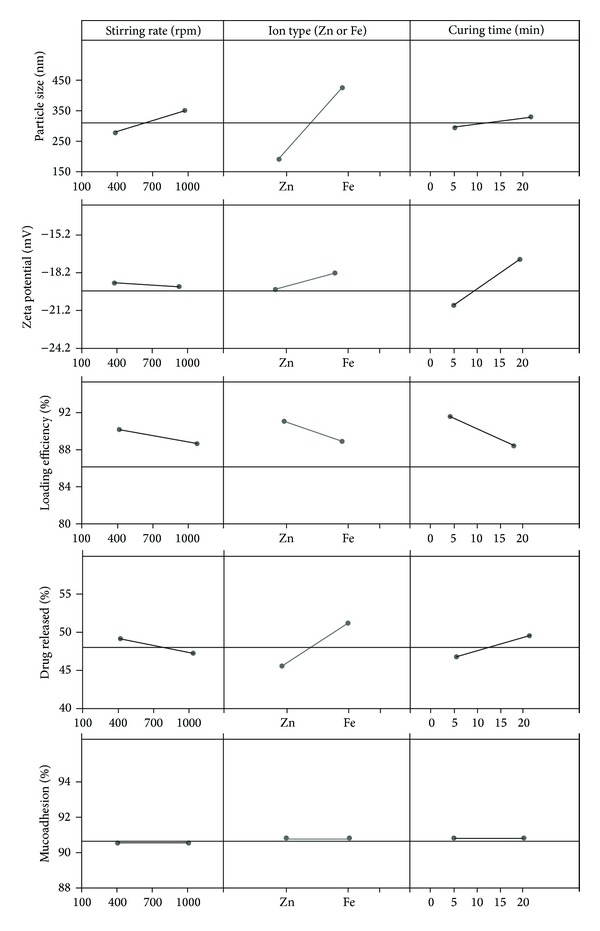
Effect of different levels of studied parameters on the particle size, zeta potential, loading efficiency drug release efficiency, and mucoadhesion ofsCT loaded P(MVEMA) nanoparticles.

Changing the ion type from Zn to Fe (Figure 2) increased the particle size of nanoparticles effectively (*P* < 0.05). While these ions have the same charge (+2), the small difference in the effective mean ionic radius of iron (78 pm) and zinc (74 pm) [49] may cause cross-linking of the polymer with Fe^+2^ more effectively than Zn^+2^. This may be the reason of greater particle size of nanoparticles prepared by Fe^+2^ compared to Zn^+2^. Figure 2 shows the other two parameters had no significant effect on the particle size of the nanoparticles (*P* > 0.05). However, increasing the stirring rate increased the particle size of nanoparticles. This may be due to increased contacts between P(MVEMA) nanoparticles and strong adhesion properties of P(MVEMA) polymer aggregated the nanoparticles.

### 3.2. Zeta Potential of Nanoparticles

Zeta potential of the nanoparticles varied between −14.6 and −22.3 mV (Table 3). The contribution effect of different studied parameters on the zeta potential of the nanoparticles is shown in Figure 1. This figure shows the most effective factor on zeta potential of nanoparticles was the curing time (*P* < 0.05) and also to a lower extent the interaction of the stirring rate and ion type (RI). Figure 2 shows that increasing the curing time increased the value of zeta potential (or decreased its absolute value) (*P* < 0.05). The prolongation of curing time caused more covalent bonds and electrical contacts between positively charged zinc or iron ions with P(MVEMA) containing COO^−^ groups with a negative charge. In fact, with increasing the cross-linking reaction of the polymer more COOH groups of the polymer are neutralized by the positive charged ions. Therefore, increasing the curing time decreased the negative charge of the nanoparticles. The decrease in the magnitude of the zeta potential suggested that P(MVEMA) chains partially shielded electric charges of the COOH groups of the polymer chains in nanoparticles. Sakuma et al. [50, 51] also reported a similar effect for poly(*N,N*-l-lysinediylterephthaloyl) microcapsules. Changing the levels of other studied variables, the ion type and stirring rate, did not have significant effect on the zeta potential (Figure 2) (*P* > 0.05).

### 3.3. Loading Efficiency of sCT in the Nanoparticles

Loading efficiency of sCT in the nanoparticles changed between 87 and 93% (Table 3). Analysis of data in Table 3 revealed that the most effective factor on drug loading efficiency was the curing time (Figure 1). Figure 2 shows almost that changing all the studied parameters (stirring rate, ion type, and curing time) from level 1 to level 2 decreased the drug loading in nanoparticles, but it was not statistically different (*P* > 0.05). The reason for reduction of drug loading efficiency by changing the ion type from Zn^+2^ to Fe^+2^ may be described by the changes in the particle size of the nanoparticles. Increasing the nanoparticles size by changing the ion type (Figure 2) is believed to be due to the fact that the higher aggregation of polymer together with the connection to the cross-linking agent caused to fill the spaces between the polymer and the ions or close the open spaces of the polymer chain and thickening of nanoparticles wall. In fact, the overall rigidity of the polymer chain increased and consequently the polymer became less permeable. This in turn could result in decreasing the drug loading efficiency. Moreover, the higher cross linking ability of Fe^2+^ may be the cause of lower loading efficiency in nanoparticles prepared by the Fe^2+^ ions. According to the amino acids sequences of the sCT, this drug has a partial positive charge (2 Lys^+^ amino acids, 1 Arg^+^ amino acid, 1 Glu^−^ amino acid) [52] and therefore the charge density of Fe^2+^ or Zn^2+^ may result in a slight repulsion between sCT and the ions. This may be the reason for loading efficiency less than 100% in the nanoparticles.

### 3.4. *In Vitro* Release of sCT from Nanoparticles

**Figure 3:**
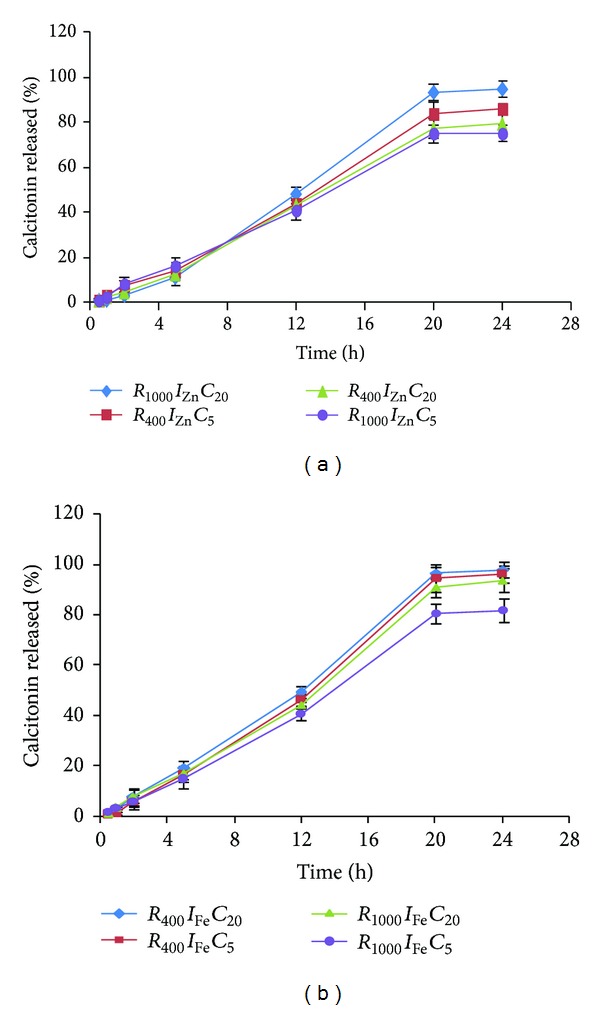
sCT release profiles from P(MVEMA) nanoparticles containing (a) Zn or (b) Fe ion (*n* = 3).

Figure 3 shows the release profiles of sCT for different studied formulations. This figure shows that release profile of the most formulations lasted for prolonged time and released approximately 80–90% of the loaded drug during 24 h. However, *R*_400_ *I*_Fe_ *C*_20_ and *R*_400_ *I*_Zn_ *C*_20_ had the highest and the lowest drug release efficiency, respectively (Table 3).  Table 4 shows the correlation coefficients of different release profiles of all formulations which better fit with a zero-order kinetic model.

The prolonged release of sCT from different nanoparticles may be attributed to the biodegradability of the polymer. However, the high concentration of the polymer solution and cross-linking agent may be responsible for the sustained release property of nanoparticles. This conclusion is consistent for both ion types (Figure 3). Drug release after about 24 h from Zn containing nanoparticles (Figure 3(a)) was almost similar to Fe ones (Figure 3(b)).

Figure 1 shows that although drug release was almost more affected by the ion type but it was not significant (*P* > 0.05). Changing the two other studied parameters, that is, ion type and curing time, from level 1 to level 2 increased the drug release from nanoparticles but not significantly (Figure 2) (*P* > 0.05). As mentioned earlier Fe^2+^ ions produced bigger particle size than Zn^2+^ ions (Figure 2). This aggregation probably expelled the trapped drug out of the nanoparticles and drug release was enhanced and consequently RE_24_% was increased (Figure 2).

### 3.5. Mucoadhesive Properties of Nanoparticles

Figure 1 shows that the most effective factor on the mucoadhesive properties of nanoparticles was the interaction of stirring rate and theion type (RI) and at a less extent the interaction of the ion type and curing time (IC). As the process of mucoadhesion is a consequence of interactions between the mucin and mucoadhesive polymer of the nanoparticles, it is greatly dependent on the polymer structure including its charge. Figure 2 indicates that none of the studied parameters had significant effect on the mucoadhesion of the nanoparticles (*P* > 0.05) and this property in all formulations was similar. In fact, mucoadhesion of all formulations was 90.6-90.7% and their differences were negligible. This is due to the constant concentration of the mucoadhesive polymer used in the production of nanoparticles.

There are many types of anionic polymers with mucoadhesive properties like poly(acrylic acid) Carbopol 980, Carbopol 974P, Carbopol 971P, polycarbophil, poly(methacryl acid) sodium salt, sodium alginate, sodium carboxymethylcelullose, sodium hyaluronate (highly polymerized grade). Some years ago, Peppas and Buri [53] analyzed the existing data and theories relevant to mucoadhesive polymers. They came to the conclusion that a number of polymer characteristics are necessary for mucoadhesion which can be summarized as follows: (i) strong hydrogen-bonding groups (–OH, –COOH), (ii) strong anionic charges, (iii) high molecular weight, (iv) sufficient chain ‘flexibility, (v) surface energy properties favouring spreading onto mucus. In contrast, negative charge and hydrogen-bonding capabilities are common to presently known mucoadhesives, but should not a priori be generalized. Instead, positively charged polymeric hydrogels could possibly develop additional molecular attraction forces by electrostatic interactions with negatively charged mucosal surfaces. In the present study as the concentration of the mucoadhesive polymer used in production of nanoparticles was constant the mucoadhesion of nanoparticles was not affected by none of the studied variables (*P* > 0.05). However, this test was carried out to prove the mucoadhesive property of the nanoparticles.

### 3.6. Optimization of the Production Process of sCT Loaded Nanoparticles

In many formulations, not just pharmaceutical in nature, it is necessary to balance several different measures of quality in order to find the best overall product. Changes in the formulation to improve one property may have a deleterious impact on another property. The process of finding the best compromise has been more rigorous by the process of desirability optimization, to produce numerical value of a desirability function.

Computer optimization process by Design Expert software and a desirability function determined the effect of the levels of independent variables on the responses. The constraint of particle size was 166.5 ≤ *Y*_1_ ≤ 553.8 nm while targeting the particle size on minimum: for zeta potential it was −22.3 ≤ *Y*_2_ ≤ −14.6 mV while the target was set on maximum of zeta potential absolute values, for drug loading efficiency the constraint was 87 ≤ *Y*_3_ ≤ 93% with the goal set at the range of obtained data (Table 3), the drug release percent after 24 h, or RE_24_% constraint was 42.2 ≤ *Y*_4_ ≤ 54.8% with the target set at the maximum values of the obtained data of Table 3 and for mucoadhesive percent constraint was 90.6 ≤ *Y*_5_ ≤ 90.7 with the target set at the maximum. Considering the data in Table 3 optimization was carried out by Design Expert software and the optimized formulation was suggested as *R*_500_ *I*_Zn_ *C*_5_,that is, using stirring rate of 500 rpm, Zn ion as the cross-linking agent, and curing time of 5 min. The predicted and actual values of responses and their standard errors are recorded in Table 5.

Table 5 shows the actual results were in close accordance with the predicted values by the software.

### 3.7. Morphological Studies

**Figure 4:**
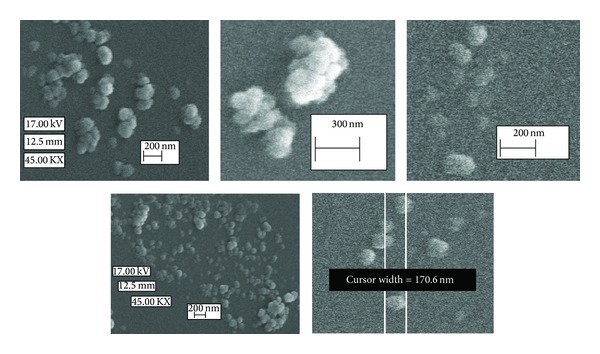
SEM micrographs of the optimized nanoparticles of sCT.

Morphology of the optimum formulation was studied by SEM (Figure 4). The SEM photographs show smooth and compact structure of nanoparticles. In general, the nanoparticles are obviously discrete some spherical and some irregularly shaped and the scale bar of the graphs confirms that the particle sizes measured by the Malvern Nanosizer instrument (Table 3) were comparable with SEM results. However, slightly aggregation of nanoparticles may be due to high bioadhesive properties of P(MVEMA) polymer.

### 3.8. SDS-PAGE Studies

**Figure 5:**
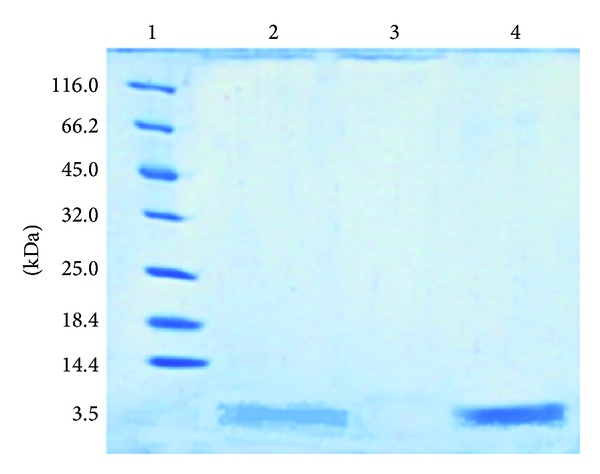
A 20% SDS-PAGE slab gel stained with Coomassie showing (1) unstained molecular weight protein markers: 116.0 kDa; *β*-galactosidase (*E. coli*), 66.2 kDa; bovine serum albumin (bovine plasma), 45.0 kDa; ovalbumin (chicken egg white), 35.0 kDa; lactate dehydrogenase (porcine muscle), 25.0 kDa; REase Bsp981 (*E. coli*), 18.4 kDa; *β*-lactoglobulin (bovine milk), 14.4 kDa; lysozyme (chicken egg white); (2) 24 h release medium of the optimized sCT nanoparticles (58.1 *μ*g mL^−1^); (3) blank sample of 24 h release medium of the optimized nanoparticles but without sCT; and (4) standard sample of sCT solution (250 *μ*g mL^−1^).

The stability of sCT during production and release test after 24 h was studied by SDS-PAGE test. The results of representative samples and unstained molecular weight protein markers are shown in Figure 5. The protein marker samples are distinctly separated and seen in 6 bands. Also, according to concentration of the implanted samples on the gel, an obvious band and a band with lower resolution were detected for standard sample (with 250 *μ*g mL^−1^ concentration) and experimental sample of 24 h release medium of the optimized formulation with 58.1 *μ*g mL^−1^ concentration.

### 3.9. Pharmacological Effect of sCT Nanoparticles

The *in vivo* pharmacological action of the prepared sCT nanoparticles was evaluated by pulmonary administration of the dispersion to male Wistar rats. As reference formulations, *iv *injection of sCT solution (5 *μ*g kg^−1^), pulmonary sCT solution (25 *μ*g kg^−1^), and blank nanoparticles dispersion (without sCT) were also included in the study. The pulmonary dose of sCT in the form of nanoparticles dispersion was 25 *μ*g kg^−1^. Considering that the rats weighted 185–330 g and their mean weight was 257.5 g, the mean dose of sCT administered via pulmonary route was 6.4 *μ*g and via *iv* injection it was 1.3 *μ*g. The final formulation of nanoparticles of sCT administered to rats consisted of 250 mg of P(MVEMA), 125 mg ZnSO_4,_ and 2.5 mg sCT in 7.5 mL deionized water. In other words, the ratio of P(MVEMA) to sCT in the nanoparticles dispersion administered to rats was 100 : 1. Consequently the mean mass of polymer administered to each animal at the sCT dose tested was 640 *μ*g in pulmonary and 130 *μ*g in *iv* injection route.

**Figure 6:**
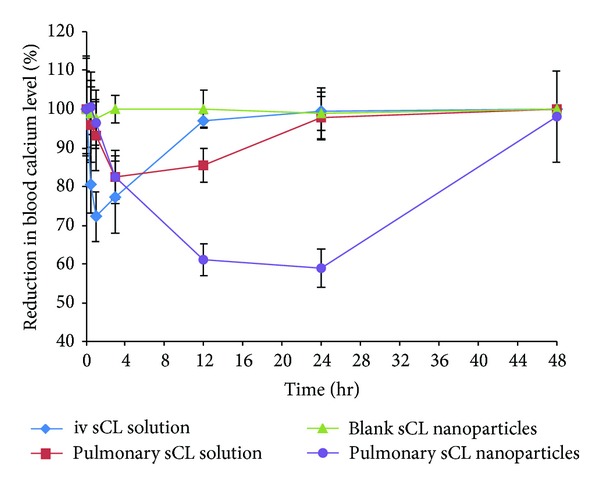
Comparison in reduction of blood calcium levels after administration of sCT in different groups of rats (*n* = 7, sCT dose of *iv *solution = 5 *μ*g kg^−1^, pulmonary dose of sCT in the form of nanoparticles or solution = 25 *μ*g kg^−1^).

The results of blood calcium levels after sCT administration in different groups are summarized in Figure 6. This figure shows pulmonary administration of sCT nanoparticles exhibited superior hypocalcemic effect compared to its pulmonary solution and *iv *administration. After pulmonary administration of sCT nanoparticles, the blood calcium levels decreased to 59% of normal level and this lasted until 24 h and a significant difference in the blood calcium concentration was observed with other groups for 24 h. In contrast, the hypocalcemic effect in the rats receiving pulmonary sCT solution persisted for 3 h, which is approximately similar to that caused by *iv* injection of sCT solution which lasted for 1 h. A possible explanation for the prolongation pharmacological effect of P(MVEMA) nanoparticles is that the nanoparticles dispersion was more viscous than the sCT solution even compared at the same concentration. The prolonged and controlled release of sCT from nanoparticles along with the mucoadhesion of this dispersion may partly contribute to the prolongation of the residence time of the nanoparticles in the pulmonary tract, leading to more effective absorption. In conclusion, the mucoadhesion and sCT release properties from the system are key factors in promoting the absorption of sCT in the lungs. This result agrees well with previous reports [54–57]. We should also pay attention to stability of peptide drugs in the pulmonary tract. The peptide drugs are susceptible to enzymatic or chemical degradation. The P(MVEMA) nanoparticles can also protect sCT against degradation in the pulmonary tract. It has also been reported that some polymers such as poly(acrylic acid) derivatives and chitosan were able to inhibit the activities of the proteolytic enzymes [57, 58].

The minimum blood calcium levels, the duration of hypocalcemia, and the area under the reduction of blood calcium levels after administration of sCT solution and sCT nanoparticles dispersion are summarized in Table 6. sCT was rapidly eliminated from the plasma after its intravenous injection in rats. The concentration of sCT in plasma decreased gradually after pulmonary administration and after 24 hours decreased the blood calcium level to approximately 59 ± 5%, but after 48 hours it reached back to baseline. In contrast, the concentration of sCT in plasma decreased rapidly after intravenous administration.

Finally, the overall pharmacological effect of pulmonary administered sCT nanoparticles was evaluated by calculating the areas above the curve (AAC) of reduced blood calcium levels up to 48 h. The results are seen in Table 7. To consider the dose of the sCT in the *iv* and pulmonary administration, the normalization was carried out by dividing the AAC to the administered dose in different routes (Table 7). This table indicates the pulmonary sCT nanoparticles caused 2.76 times higher effect (AAC/dose) than that of pulmonary administered free sCT solution. However, no significant difference (*P* > 0.05) was seen in pharmacological action (AAC/dose) between the pulmonary sCT solution (25 *μ*g kg^−1^) and *iv* (5 *μ*g kg^−1^) administered solution of this drug (Table 7).

The relative short duration of action of pulmonary solution of sCT may be the result of rapid elimination of drug by mucociliary clearance and/or drug degradation by pulmonary peptidases [59]. On the other hand, prepared sCT nanoparticles significantly prolonged the hypocalcemic effect of sCT with approximate pharmacological activity relative to *iv* route of 119% (Table 7).

Moreover, partial protection against enzymatic degradation is expected due to the protecting effect of the nanoparticles. The higher efficacy and longer duration of hypocalcemic effect of the prepared nanoparticles could be the result of the higher mucoadhesive properties of the P(MVEMA) polymer as mentioned earlier.

Comparison of the results of the current study with previous findings should be made with caution because of the differences in the *in vivo* model, dose, sCT type, administration method, and evaluation procedure. However, P(MVEMA) polymer and its derivative should be considered as potentially safe and effective polymer for enhancing the pulmonary delivery of peptide drugs. Zinc ion (cross linking reagent) is generally regarded as biologically compatible and safe material as compared to the other ions. Additionally, formulation of sCT nanoparticles offers an appropriate size for avoiding alveolar macrophage clearance and promoting transepithelial transport in the alveolar region.

## 4. Conclusion

The pulmonary sCT nanoparticles of P(MVEMA) were prepared for increasing hypocalcemic action and enhancing the duration of their hypocalcemic effect. The cross-linking ion used in their preparation caused reproducible results. The stirring rate, ion type, and curing time were detected as effective variables in production of the sCT nanoparticles. The best formulation of the sCT nanoparticles was prepared by stirring rate of 500 rpm, ion type of Zn as cross-linking agent, and the curing time of 5 min. We demonstrated that the pharmacological availability of pulmonary sCT nanoparticles was approximately 3-fold higher than that of pulmonary sCT solution in rats. Furthermore, it was confirmed that the elimination rate of sCT loaded nanoparticles from the lungs was decreased significantly due after pulmonary administration compared to that of the free sCT. The sCT loaded nanoparticles resulted in a pronounced hypocalcemic effect for at least 24 h and a corresponding reduction in plasma calcium level of approximately 59%. Inhaled sCT nanoparticles improved hypocalcemic control over that of intravenous sCT. However, further *in vivo* pharmacokinetic and toxicity studies should be performed to check the effectiveness and safety of the developed formulation. Moreover; future studies will establish whether or not this suggestion will be verified in the marketplace.

## Figures and Tables

**Table 1:** Studied variables in factorial design used in preparation of sCT loaded in P(MVEMA) nanoparticles.

Studied variables	Levels	
	II	I
Stirring rate (rpm)	400	1000
Ion type	Zn^2+^	Fe^2+^
Curing time (min)	5	20

**Table 2:** Composition of different formulations of sCT loaded P(MVEMA) nanoparticles designed by a two levels factorial design.

Formulation code	Stirring rate (*R*) (rpm)	Ion type (*I*)	Curing time (*C*) (min)
*R*_1000_ *I*_Fe_ *C*_5_	1000	Fe	5.00
*R*_400_ *I*_Fe_ *C*_5_	400	Fe	5.00
*R*_400_ *I*_Zn_ *C*_20_	400	Zn	20.00
*R*_1000_ *I*_Zn_ *C*_5_	1000	Zn	5.00
*R*_1000_ *I*_Zn_ *C*_20_	1000	Zn	20.00
*R*_400_ *I*_Zn_ *C*_5_	400	Zn	5.00
*R*_1000_ *I*_Fe_ *C*_20_	1000	Fe	20.00
*R*_400_ *I*_Fe_ *C*_20_	400	Fe	20.00

**Table 3:** Physicochemical properties of P(MVEMA) nanoparticles loaded with sCL.

Formulation code	Particle size (nm)	Zeta potential (mV)	Drug loading efficiency (%)	Drug release efficiency after 24 h (RE_24_ %)	Mucoadhesive efficiency (%)
*R*_1000_ *I*_Fe_ *C*_5_	449.4 ± 3.8	−21.8 ± 1.8	90.0 ± 3.5	44.8 ± 2.7	90.64 ± 0.04
*R*_400_ *I*_Fe_ *C*_5_	381.4 ± 16.1	−19.7 ± 2.4	88.5 ± 3.0	52.3 ± 4.9	90.66 ± 0.04
*R*_400_ *I*_Zn_ *C*_20_	227.4 ± 6.6	−19.1 ± 1.0	91.0 ± 1.0	42.3 ± 1.5	90.64 ± 0.04
*R*_1000_ *I*_Zn_ *C*_5_	206.8 ± 10.8	−19.0 ± 1.4	92.0 ± 3.0	42.6 ± 0.9	90.71 ± 0.05
*R*_1000_ *I*_Zn_ *C*_20_	189.2 ± 8.4	−18.3 ± 1.1	86.0 ± 2.0	49.2 ± 2.5	90.65 ± 0.06
*R*_400_ *I*_Zn_ *C*_5_	166.5 ± 1.0	−22.3 ± 0.6	93.0 ± 0.5	46.2 ± 0.5	90.65 ± 0.03
*R*_1000_ *I*_Fe_ *C*_20_	553.8 ± 20.5	−17.4 ± 0.4	87.0 ± 2.0	51.0 ± 2.3	90.65 ± 0.04
*R*_400_ *I*_Fe_ *C*_20_	349.6 ± 6.9	−14.6 ± 0.8	88.0 ± 3.5	54.4 ± 2.9	90.66 ± 0.06

**Table 4:** Correlation coefficients of different kinetic models obtained by curve fitting method to sCT release data from different nanoparticles.

Formulation code	First-order	Zero-order	Higuchi model
*R*_1000_ *I*_Fe_ *C*_5_	0.921	0.986	0.952
*R*_400_ *I*_Fe_ *C*_5_	0.936	0.986	0.952
*R*_400_ *I*_Zn_ *C*_20_	0.956	0.987	0.959
*R*_1000_ *I*_Zn_ *C*_5_	0.935	0.983	0.967
*R*_1000_ *I*_Zn_ *C*_20_	0.967	0.985	0.946
*R*_400_ *I*_Zn_ *C*_5_	0.969	0.986	0.956
*R*_1000_ *I*_Fe_ *C*_20_	0.925	0.985	0.950
*R*_400_ *I*_Fe_ *C*_20_	0.955	0.986	0.958

**Table 5:** Comparison of the Design Expert predicted and actual values of responses studied in sCT loaded P(MVEMA) nanoparticles.

Response	Particle size (nm)	Zeta potential (mV)	Drug loading efficiency (%)	Release efficiency (RE_24_ %)	Mucoadhesive efficiency (%)
Predicted	188.3	−21.0	92.7	45.1	90.67
Actual	196.1 ± 6.9	−19.7 ± 1.7	92.2 ± 2.5	49.5 ± 2	90.66 ± 0.04
Error %	4.1	6.2	0.5	9.9	0.01

**Table 6:** Duration of hypocalcemic effect and maximum reduction in blood calcium levels after pulmonary and iv administration of sCT solution and sCT nanoparticles dispersion. Results are expressed as mean ± SE of seven rats.

Studied parameter	Intravenous injection of sCT solution	Pulmonary administration	
		sCT solution	sCT nanoparticles dispersion
Dose (µg kg^−1^)	5	25	25
Blood calcium level compared to normal level (%)	72 ± 7.5	82.5 ± 9.5	59 ± 5
Duration of hypocalcemia (h)	0.5–1	3-4	12–24
AAC_0–48_(% h)	211.0 ± 63.0	302.1 ± 100.9	1259.9 ± 147.2

**Table 7:** Pharmacodynamics of sCT after iv and pulmonary administration to rats.

Formulation	Dose (µg kg^−1^)	AAC_0–48 h_ (% h)	AAC_0–48 h_/dose (% h kg µg^−1^)	Pharmacological activity relative to *iv* route (%)
*iv* solution of sCT	5	211.05 ± 63.03	42.21 ± 12.61	—
Pulmonary sCT solution	25	302.08 ± 100.93	12.08 ± 4.04^*¥*^	28.63 ± 9.56
Pulmonary blank nanoparticles	25	25.10 ± 5.56	1.00 ± 0.22	2.4 ± 0.53
Pulmonary sCT nanoparticles	25	1259.95 ± 147.19	50.40 ± 5.89^∗*¥*^	119.4 ± 13.95

*Significant difference (*P* < 0.001) between iv solution of sCT and pulmonary sCT nanoparticles, ^¥^significant difference (*P* < 0.001) between pulmonary sCT solution and pulmonary sCT nanoparticles.
